# Maltreatment-Exposure Screening and Adolescent Mental Health: An Evidence-Based Medicine Perspective on Accuracy and Fairness

**DOI:** 10.1007/s40653-025-00767-1

**Published:** 2025-11-08

**Authors:** Jae Wan Choi, Joseph R. Cohen

**Affiliations:** Department of Psychology, University of Illinois, 603 E. Daniel Street, Champaign, IL 61820 USA

**Keywords:** Child maltreatment, Trauma-informed screening, Statistical discrimination, Statistical calibration, Statistical fairness

## Abstract

Universal trauma-informed screening is increasingly recommended across pediatric settings, yet it remains unclear which forms of adversity most accurately and equitably identify youth at risk for psychopathology. In response, the current study tested whether defining maltreatment by cumulative quality (i.e., number of subtypes) versus cumulative frequency (i.e., number of experiences regardless of subtypes), as well as potential differences within each subtype, yielded more accurate and fair predictions of trauma-related psychological distress. Participants included 839 adolescents from the LONGSCAN study (50.5% identified as female; 55.2% identified as Black) who completed self-reports of maltreatment exposure and psychological distress at ages 12, 16, and 18. Statistical accuracy was assessed via statistical discrimination (area-under-the-curve [AUC]) and calibration (Spiegelhalter’s Z; calibration curves), while statistical fairness was evaluated through differences in accuracy across adolescent subgroups (i.e., gender; race/ethnicity). Results across cumulative risk models revealed equivocal results regarding accuracy, while demonstrating important patterns of unfairness. In contrast, analyses by maltreatment subtype identified emotional abuse quality and frequency as both accurate (AUC > 0.64; non-significant Spiegelhalter’s Zs; 95% confidence intervals for intercept and slope included 0 and 1, respectively) and statistically fair in identifying concurrent risk for trauma-related distress. Cumulative frequency and emotional abuse frequency also demonstrated accuracy and potential fairness in predicting risk at age 16, whereas no indicators demonstrated clinical utility at age 18. Overall, findings suggest that emotional abuse may be useful in screening for concurrent psychological distress, while adding to the literature questioning the continued reliance on cumulative ACEs scores in clinical and public health contexts.

**Supplementary Information:**

The online version contains supplementary material available at https://doi.org/10.1007/s40653-025-00767-1.

## Introduction

Nearly half of all youth living in the United States are exposed to adverse childhood experiences (ACEs; Sacks & Murphey, 2018), which include physical, emotional, and sexual maltreatment as well as other family adversities (e.g., substance using caregivers; Hughes et al., 2017; Fitzgerald & Gallus, 2025). Adversity exposure is a robust transdiagnostic risk factor for multiple forms of psychopathology (e.g., post-traumatic stress disorder [PTSD]; Alisic et al., 2014), as youth exposed to adversity are twice as likely to develop mental health disorders compared to their non-exposed counterparts (Lewis et al., 2019). As adolescence, relative to adulthood, is a sensitive period for developing trauma-related distress (e.g., Infurna et al., 2016), preventative universal screening for childhood adversity exposures is now recommended across settings that serve adolescence (e.g., Dube, 2018). Universal screening for childhood adversity carries the potential to positively impact adolescent mental health, including early identification of at-risk youth who may otherwise go unrecognized and untreated, potential prevention of downstream healthcare expenses, increased awareness of youth’s adversity exposures and related symptoms, and increased collaboration between mental health providers and additional supports (e.g., child welfare) to provide youth with evidence-based trauma-informed treatment (e.g., Merikangas et al., 2011; Butcher et al., 2020). Thus, there is considerable attention for developing protocols that accurately and equitably capture the mental health risk associated with exposure to childhood adversity-exposure.

Youth are more likely to experience multiple adversities rather than a single incident (Turner et al., 2010; Finkelhor et al., 2013). Polyvictimization, in turn, has been linked to greater risk for chronic patterns of psychological distress (e.g., Finkelhor et al., 2007). Reflecting these patterns, most preventive screening protocols assess adversity exposure in terms of cumulative quality of exposure – that is, the aggregate number of different adversity subtypes experienced (Kelly-Irving & Delpierre, 2019). While this approach is supported by evidence showing dose-response associations between adversity and mental health outcomes, it rests on the assumption that each adversity subtype contributes equally to psychological risk. Recent research has challenged this assumption, however, highlighting that some adversities may be more harmful than others. For example, research by Atzl and colleagues (2019) and Negriff (2020) found that maltreatment events (i.e., physical, sexual, or emotional abuse, physical or emotional neglect) were stronger predictors of psychological distress compared to other family adversities (e.g., caregiver substance abuse). Moreover, variation across maltreatment subtypes has also been observed, with certain forms (e.g., emotional abuse; Cecil et al., 2017) emerging as particularly potent risk factors for trauma-related psychopathology among youth. Together, these findings suggest that screening protocols may benefit from prioritizing maltreatment exposure and differentiating among maltreatment subtypes, which may improve risk detection beyond indexing cumulative quality of exposure or broader household dysfunction alone.

Prior to prioritizing certain maltreatment subtypes, however, it may be necessary to examine other maltreatment facets beyond *quality* (i.e., the number of subtypes experienced) which may confer adolescent mental health risk. For example, Vachon et al. (2015) found that the *frequency* (i.e., the number of incidents reported) of maltreatment, regardless of the subtypes experienced, predicted greater levels of psychological distress. Ellenbogen et al. (2013) also highlighted the importance of examining frequency when predicting maladaptive effects of maltreatment exposure among adolescents, while further demonstrating that frequency of maltreatment was a robust predictor of maladaptive behaviors, even after controlling for other facets of maltreatment including maltreatment quality, chronicity (i.e., span of time over which the maltreatment took place), and age of onset (i.e., the age at which one was first exposed to maltreatment). These collective findings may suggest that past research concerning certain maltreatment subtypes being uniquely deleterious (e.g., emotional abuse; Infurna et al., 2016) may be due to them being a more frequent form of maltreatment as opposed to the specific nature of that maltreatment experience. Translationally, findings that maltreatment frequency may confer greater risk than maltreatment subtypes suggest that current practices of indexing cumulative risk via quality (e.g., a series of yes/no questions on a subset of maltreatment experiences) rather than the frequency of adversity-exposure (i.e., how many times youth have been exposed to maltreatment) is not congruent with population health findings.

In addition to identifying which types and dimensions of adversities may be particularly salient in predicting youth mental health, it is also critical to establish the statistical accuracy of screening protocols across different contexts. Drawing on the evidence-based medicine framework – which emphasizes the translation of research into decision making within medical and other applied settings (Youngstrom et al., 2017) – testing both *statistical discrimination* and *statistical calibration* is necessary to provide a comprehensive assessment of prediction model performance (Lindhiem et al., 2020). First, statistical discrimination pertains to a model’s ability to accurately distinguish between those with and without psychopathology and is suggested to be particularly important in making clinical decisions at the individual level (Cohen & Choi, 2022; Cohen & Stutts, 2023). To date, statistical discrimination has only been examined with regard to cumulative trauma models, which have produced poor results (e.g., Meehan et al., 2022). For example, Baldwin and colleagues (2021) examined the area under the curve (AUC) of total ACEs scores based on subtypes and found that the overall score had poor accuracy in predicting prospective psychopathology in youth. This may be because the overall ACEs score includes experiences that are less associated with mental health symptoms (e.g., household dysfunctions; Negriff, 2020) and the exclusion of such items may lead to improved accuracy. Another possibility is that these studies did not account for differences in frequency within certain adversity subtypes. Thus, determining the statistical properties of each adversity index from a multi-faceted perspective may help to provide a more accurate prediction of mental health outcomes.

Another important facet of statistical accuracy that has received less attention is *calibration*. Calibration refers to a model’s accuracy regarding the agreement between observed and predicted outcomes (Van Calster et al., 2019). In other words, calibration assesses whether the probabilistic prediction of an event matches the true underlying probability of the event (Lindhiem et al., 2020) and is particularly important in making decisions at the community level to help inform public health programming (Cohen & Choi, 2022; Cohen & Stutts, 2023). Although related, calibration and discrimination are orthogonal constructs that assess distinct components of accuracy (Lindhiem et al., 2020). Specifically, while discrimination refers to how well a tool correctly classifies someone as at-risk or not at-risk, calibration refers to how much someone at-risk is likely to present with that outcome. Thus, it is possible for a prediction model to have perfect discrimination, but not useful calibration properties (and vice versa). For instance, a model that correctly forecasts that 51% of adolescents with PTSD have concentration difficulties, and 49% of adolescents that do not have PTSD have concentration difficulties, is accurate from a *discrimination* perspective, but is not well-calibrated, because there is such little differentiation between the risk categories. Although the discrimination of ACEs screening has been examined (Baldwin et al., 2021; Meehan et al., 2022), examinations of calibration have been limited to one cross-sectional study (Cohen & Choi, 2022), in which they found that total scores on the ACEs showed acceptable levels of calibration in predicting PTSD, depression, and externalizing disorders in adolescents. Extending these findings to indices of frequency, as well as individual maltreatment subtypes, can help inform public health screening initiatives where adversity screening is most common (Anda et al., 2020).

Finally, in addition to accuracy, it is equally important to test *statistical fairness*. Statistical fairness refers to whether the measure of risk predicts outcomes with similar levels of accuracy across subpopulations (Berk et al., 2021). A lack of fairness could introduce bias in the utilization of maltreatment screening and inequality in the allocation of necessary preventive resources for youth at risk (Senior et al., 2021). Previous research has highlighted that the association between maltreatment exposure and psychopathology varies as a function of race/ethnicity (e.g., Woods-Jaeger et al., 2021), which may lead to less accurate predictions of psychopathology among adolescents who identify as racial/ethnic minority compared to their White counterparts (Bernard et al., 2021). Similarly, research has also suggested that the association between maltreatment exposure and mental health issues may differ across males and females (e.g., Asscher et al., 2015), highlighting potential gender biases that will emerge when screening for maltreatment experiences. Research within the context of ACEs has shown that while the total ACEs score shows adequate discrimination and acceptable calibration for concurrent mental health outcomes across the full sample, examination of subpopulations revealed patterns of statistical unfairness (Cohen & Choi, 2022). As such, it is necessary to test whether certain maltreatment experiences may be contributing to, or immune from, potential disparities in mental health risk prediction.

The present study sought to highlight the potential promise and pitfalls of focusing on maltreatment-exposure to index adolescent mental health risk by testing the following aims. First, we examined the statistical accuracy *and* fairness of indexing cumulative mental health risk based on (a) the number of maltreatment subtypes experienced across the lifespan (i.e., cumulative quality) and (b) the frequency of maltreatment experiences across the lifespan (i.e., cumulative frequency). We then extended these aims to individual maltreatment subtypes to examine whether the number of different acts (i.e., quality) or the number of incidents (i.e., frequency) best indexed risk. Our decision to focus on maltreatment quality was to reflect its high prevalence in translational screening efforts (e.g., Anda et al., 2020), while maltreatment frequency was informed by research demonstrating frequency to better index trauma-related outcomes compared to subtypes, chronicity, and age of onset (e.g., Ellenbogen et al., 2013). These analyses were conducted across (a) concurrent and prospective mental health outcomes and (b) adolescent subpopulations defined by gender and race/ethnicity.

Overall, following previous research (e.g., Vachon et al., 2015; Ellenbogen et al., 2013), we hypothesized that cumulative frequency would provide an incrementally valid prediction of diagnostic status relative to assessing for cumulative quality. Regarding maltreatment subtypes, we hypothesized experiences of emotional abuse to demonstrate stronger levels of accuracy in predicting outcomes compared to other subtypes. Exploratory research was conducted to examine if quality of emotional abuse (e.g., a parent blamed a child for his or her own problems) or frequency of emotional abuse best predicted mental health outcomes. Finally, following past research that have found different associations between maltreatment exposure and adolescent mental health across gender (e.g., Asscher et al., 2015) and race/ethnicity (e.g., Cohen & Choi, 2022; Woods-Jaeger et al., 2021), we hypothesized that patterns of unfairness will emerge, such that higher scores of maltreatment exposure would overestimate the risk for poor mental health in adolescent subpopulations that identify as Male or Black.

## Methods

### Participants and Procedures

The present study utilized secondary data from the Longitudinal Studies in Child Abuse and Neglect (LONGSCAN; Runyan et al., 1998). LONGSCAN is a consortium of parallel longitudinal studies across 5 sites (i.e., East, Midwest, South, Southwest, and Northwest) to investigate the long-term antecedents and consequences of child maltreatment. While all sites used identical measures, data collection protocols, and assessment timepoints, the sampling strategies varied across locations. Specifically, the East sample consisted of low-income children who were recruited from primary healthcare clinics, and the Midwest and Northwest samples included children who were reported to Child Protection Services (CPS). Children from the South site were recruited from a High Priority Infant Program, and the Southwest sample consisted of youth who had been placed in CPS custody. Participants were interviewed every 2 years beginning at age 4. Additional information regarding the LONGSCAN study is available elsewhere (Runyan et al., 1998). The current study utilized the data collected at ages 12, 16, and 18. Adolescents with missing data at age 12 were excluded from the survey. The final sample consisted of 839 adolescents (*M*_*Age*_=12.38; *SD*_*Age*_=0.44), which was equally distributed across gender (50.5% female). As for race/ethnicity, 55.2% of the sample identified as Black, 26.2% identified as White, 6.6% identified as Hispanic, and 12.1% identified as “other”.

### Measures

#### Outcomes

**Trauma Symptom Checklist for Children (TSCC; Briere, **1996**)** At ages 12 and 16, 44 items of the TSCC were used to assess levels of trauma-related psychological distress, consisting of depression, anxiety, anger, post-traumatic stress (PTS), and dissociation subscales. Adolescents were asked to indicate the frequency of symptoms on a 4-point Likert scale, ranging from 0 (Never) to 3 (Almost all of the time). Following recommendations, adolescents with t-scores at or above 65 were considered clinically significant (Briere, 1996). Relatedly, adolescents who fell in the clinical range for at least one of the subscales were coded as having experienced a trauma-related mental health disorder. Past research has demonstrated validity of the TSCC among adolescents (Briere, 1996). In the current study, Cronbach’s alpha for the TSCC was 0.96 at both ages 12 and 16, indicating excellent reliability.

**Trauma Symptom Inventory (TSI; Briere, **1995**)** As children aged to 18 in the study, they subsequently completed 50 items of the TSI to assess prospective levels of psychological distress, consisting of depression, anxious arousal, anger/irritability, intrusive experiences, defensive avoidant, and dissociation subscales. The TSI is similar to the TSCC and is intended to provide a more developmentally sensitive assessment of trauma-related distress as one ages into adulthood. Adolescents for this scale were asked to indicate the frequency of symptoms experienced in the past 6 months on a 3-point Likert scale, ranging from 1 (*Never*) to 3 (*Often*). Following recommendations, adolescents with t-scores at or above 65 were considered clinically significant (Biere, 1995). Similar to the TSCC, adolescents who fell in the clinical range for at least one of the subscales were coded as having experienced a trauma-related mental health disorder. Validity of the TSI among youth has been demonstrated previously (Biere, 1995). In the current study, Cronbach’s alpha for the TSI was 0.97, suggesting excellent reliability.

#### Maltreatment

**Physical/Emotional/Sexual Abuse **Project-developed self-report measures of physical (15 items), emotional (18 items), and sexual (11 items) abuse were used to assess for experiences of abuse (Everson et al., 2008). The measures are comprised of stem questions asking about lifetime exposure to specific experiences, which, if endorsed, triggered follow-up questions regarding timeframe of occurrence, frequency, and perpetrators. Pre-tests with 24 youth in face-to-face interviews indicated that youth were comfortable with the structure and wording of the items, and were accurate in their reports (Knight et al., 2000). Congruent with past research (Kobulsky et al., 2018), adolescents who endorsed at least one of the items under each abuse subtype were coded as having experienced the respective subtype. In addition to a dichotomized indication of experiencing each subtype, quality of exposure was further assessed by summing the number of items endorsed under each subtype. Finally, frequency of abuse was assessed by summing the frequency of each item experienced *before elementary school* and *since starting elementary school until now*, reported on a 4-point Likert scale ranging from 0 (*never*) to 3 (*4 or more*) for physical and sexual abuse, and a 3-point Likert scale ranging from 0 (*never*) to 2 (*often*) for emotional abuse. Frequencies for physical and sexual abuse were subsequently recoded into a 3-point Likert scale, ranging from 0 (*never*), 1 (*1 time*), or 2 (*2 or more times*), to allow for a more consistent comparison across the maltreatment subtypes.

**Physical/Emotional Neglect **14 items of the Modified Multidimensional Neglectful Behavior Scale (MNBS-A; Straus et al., 1995; Dubowitz et al., 2011) were used to assess for physical (7 items) and emotional (7 items) neglect. For each item, adolescents indicated the frequency of specific experiences involving their parents while they were in elementary school, on a 4-point Likert Scale, ranging from 0 (*never*) to 3 (*a lot*). In the current study, items were reverse coded such that higher scores indicated greater levels of neglect. Similar to the frequencies for physical abuse and sexual abuse, frequencies for each neglect subscale were recoded into a 3-point Likert Scale ranging from 0 (*never* and *almost never*), 1 (*sometimes*), and 2 (*a lot*) to allow for a more accurate comparison. Additionally, each item was dichotomized to assess for the number of types of neglect experienced as either 0 (*not endorsed*) or 1 (*endorsed*). Similar to the abuse subtypes, adolescents who endorsed any of the neglect items were coded as having experienced the respective neglect subtype, while the quality of exposure to each subtype was further assessed by summing the number of items endorsed. Validity of the MNBS-A among adolescents has been demonstrated in past research (Dubowitz et al., 2011). In the current study, Cronbach’s alpha for the physical neglect and emotional neglect subscales of the MNBS-A were 0.82 and 0.81, respectively, suggesting good internal consistency.

### Data Analytic Approach

Preliminary analyses were conducted to examine potential patterns of missing data and compute descriptive statistics. Maltreatment quality and frequency were recoded to ensure that recommendations for at least 10 cases per marginal point were met (Kraemer, 1992). For example, adolescents who reported at least 5 physical abuse events (*N* = 6), were merged with adolescents who reported 7 events (*N* = 2), 9 events (*N* = 1), and 10 events (*N* = 1). Cumulative frequency of maltreatment exposure across all maltreatment subtypes was calculated as the sum across subtypes, with each subtype’s raw count divided by the number of items for that subtype to maintain a comparable scale. Recoding for all maltreatment subtypes are explained in Appendix A. Given methodological limitations of including covariates in the main analysis (Janes & Pepe, 2006; Van Calster et al., 2019), subsequent logistic regression models controlling for the effects of gender, race/ethnicity, and recruitment site were conducted to examine which maltreatment-exposure indices were related to concurrent (i.e., age 12) trauma-related psychopathology, while regression models further controlling for psychopathology at age 12 examined which indices of maltreatment were related to prospective (i.e., ages 16 and 18) outcomes.

We first examined statistical discrimination via receiver operating characteristic (ROC) curves, in which indices that demonstrated at least a significant (i.e., *p* ≤.05) medium effect size (AUC ≥ 0.64) were considered clinically useful (Rice & Harris, 2005). Subsequently, DeLong’s test was conducted among indices that demonstrated clinical utility to test whether specific indices of maltreatment better classified risk (Youngstrom, 2014). Next, calibration was tested by using Spiegelhalter’s z test and calibration curves. Spiegelhalter’s z test is a decomposition of the Brier score, which includes components of both discrimination and calibration (Lindhiem et al., 2020). As a result, the Spiegelhalter’s z test provides an overall assessment of a model’s calibration, with significant scores (i.e., *p* <.05) indicating miscalibration. In addition to the Spiegelhalter’s z, calibration curves, generated from calibration plots representing the relation between the estimated risk (x-axis) and the observed proportion of events (y-axis; Van Calster et al., 2019), were also examined. Calibration curves were created by smoothing calibration plots via smoothing techniques (e.g., loess algorithm), where a 45˚ line represents perfect calibration. The intercept (i.e., calibration-in-the-large; CIL) and slope of generated calibration plots were evaluated to examine the calibration of each index. Specifically, the CIL has a target value of 0 and assesses for overestimation (CIL < 0) or underestimation (CIL > 0), while the slope has a target value of 1 and evaluates whether estimated risks are too extreme (slope < 1; i.e., too high for youth who are at high risk and too low for youth who are at low risk) or too moderate (slope > 1; Van Calster et al., 2019). Additionally, Emax, the maximal absolute difference between observed and predicted probabilities, was used to assess for overall calibration, with lower scores demonstrating better performance (Harrell, 2001). All calibration indices included 95% confidence intervals which were derived using bootstrap methods with 2000 bootstrap samples.

Of note, an initial series of analyses tested accuracy for each form of mental health risk (i.e., depression, anxiety, anger, pts, dissociation). However, this approach was under-powered and was remarkably similar to analyses that did have adequate power (e.g., at least 10 positive outcomes in each cell for calibration). Thus, models were collapsed into a single model for anyone scoring above threshold on any form of psychopathological risk. Appendix B summarizes the findings for specific forms of psychopathology.

Following tests of statistical accuracy, tests of statistical fairness examined whether indices of maltreatment demonstrating both discrimination and calibration accuracy at each age provided equitable predictions across adolescent gender and race/ethnic subgroups. Modeled after recent examples (e.g., Cohen & Stutts, 2023), statistical fairness for discrimination was examined by evaluating significant differences in ROC curves via DeLong’s test (Youngstrom, 2014). Relatedly, statistical fairness for calibration was evaluated by significant differences in the CILs and slopes generated via calibration curves (i.e., CILs and slope estimates that fall outside the 95% confidence intervals). Following recommendations, analyses were considered exploratory if less than 20 positive cases existed within the subpopulation of interest (Youngstrom, 2014; Kraemer, 1992). Analyses were conducted in SPSS v25.0 (IBM Corp, 2021) and in R using the pROC (Robin et al., 2011) and rms packages (Harrell et al., 2017).

## Results

### Preliminary Analyses

Descriptive statistics of the study variables, including means, standard deviations, and bivariate correlations are presented in Table 1. First, examination of outcomes revealed 41 (4.8%), 37 (4.4%), and 119 (14.2%) adolescents endorsing trauma-related psychopathology at ages 12, 16, and 18, respectively. Outcomes stratified by gender and race/ethnicity are available in Table 1. Across gender, 27 females and 14 males endorsed psychopathology at age 12, and 21 females and 16 males endorsed psychopathology at age 16, while 71 females and 47 males endorsed psychopathology at age 18. With regard to race/ethnicity, 20 adolescents identifying as Black and 11 adolescents identifying as White endorsed distress at age 12, while 18 adolescents identifying as Black and 13 adolescents identifying as White, and 59 Black and 34 White adolescents endorsed distress at ages 16 and 18, respectively. Meanwhile, among Hispanic adolescents, only 4 (7.3%), 2 (4.8%), and 5 (11.9%) adolescents reported endorsing psychopathology across the 3 waves, respectively. Due to the small number of Hispanic adolescents endorsing mental health outcomes, tests of fairness across race/ethnicity were limited to examination between adolescents identifying as Black or White.

**Table 1:** Means, standard deviations, and bivariate correlations of study variables

Variable	Total Mean (SD)	Female Mean (SD)	Male Mean (SD)	Black Mean (SD)	White Mean (SD)	1.	2.	3.	4.	5.	6.	7.	8.	9.	10.	11.	12.	13.	14.	15.
1. Cumulative Quality	1.26 (1.26)	1.25 (1.21)	1.27 (1.30)	1.23 (1.25)	1.26 (1.24)	-														
2. Cumulative Frequency	0.37 (0.70)	0.36 (0.67)	0.39 (0.72)	0.37 (0.74)	0.33 (0.53)	0.71**	-													
3. Phys. Neg. Quality	0.17 (0.62)	0.16 (0.58)	0.19 (0.66)	0.19 (0.70)	0.13 (0.37)	0.40**	0.48**	-												
4. Phys. Neg. Frequency	0.18 (0.75)	0.12 (0.58)	0.23* (0.89)	0.19 (0.83)	0.15 (0.55)	0.38**	0.54**	0.78**	-											
5. Emo. Neg. Quality	0.62 (1.20)	0.52 (1.11)	0.72* (1.28)	0.59 (1.19)	0.58 (1.01)	0.56**	0.63**	0.57**	0.51**	-										
6. Emo. Neg. Frequency	0.82 (2.01)	0.66 (1.73)	0.99* (2.25)	0.84 (2.09)	0.69 (1.56)	0.48**	0.66**	0.51**	0.58**	0.90**	-									
7. Phys. Abuse Quality	0.40 (0.92)	0.38 (0.84)	0.41 (0.99)	0.40 (0.95)	0.43 (0.95)	0.59**	0.53**	0.16**	0.13**	0.22**	0.17**	-								
8. Phys. Abuse Frequency	0.62 (1.82)	0.66 (1.81)	0.59 (1.82)	0.60 (1.81)	0.71 (1.85)	0.51**	0.57**	0.15**	0.15**	0.20**	0.15**	0.88**	-							
9. Emo. Abuse Quality	1.42 (2.33)	1.57 (2.42)	1.27 (2.21)	1.31 (2.24)	1.49 (2.34)	0.66**	0.65**	0.13**	0.13**	0.27**	0.25**	0.56**	0.53**	-						
10. Emo. Abuse Frequency	2.02 (4.37)	2.24 (4.58)	1.80 (4.13)	1.87 (4.38)	2.00 (3.71)	0.55**	0.70**	0.11**	0.12**	0.24**	0.21**	0.49**	0.52**	0.87**	-					
11. Sex. Abuse Quality	0.39 (1.13)	0.44 (1.26)	0.33 (0.97)	0.38 (1.10)	0.32 (1.06)	0.49**	0.56**	0.05	0.06	0.10**	0.11**	0.31**	0.33**	0.34**	0.34**	-				
12. Sex. Abuse Frequency	0.75 (2.73)	0.91 (3.23)	0.58 (2.09)	0.75 (2.63)	0.61 (2.59)	0.40**	0.60**	0.06	0.08*	0.07*	0.07*	0.30**	0.34**	0.33**	0.36**	0.89**	-			
13. Age 12 Psychopathology	41 (4.8)	27 (6.3)	14 (3.3)	20 (4.3)	11 (5)	0.17**	0.17**	0.04	0.04	0.08*	0.07*	0.10**	0.15**	0.19**	0.21**	0.08*	0.08*	-		
14. Age 16 Psychopathology	37 (4.4)	21 (7.4)	16 (5.7)	18 (6.1)	13 (9.2)	0.11**	0.14**	0.11**	0.03	0.07	0.05	0.12**	0.12**	0.14**	0.12**	0.12**	0.13**	0.11**	-	
15. Age 18 Psychopathology	119 (14.2)	71 (21.1)	47 (16.2)	59 (17.1)	34 (20.7)	0.09*	0.13**	0.01	− 0.01	0.03	0.01	0.09*	0.11**	0.12**	0.12**	0.15**	0.12**	0.05	0.20**	-

Note: Cumulative Quality = Total number of maltreatment subtypes endorsed at Baseline (Age 12); Cumulative Frequency = Sum of the frequency of each subtype divided by the number of items used to assess each subtype, respectively, endorsed at Baseline; Phys. Neg. Quality = Number of items endorsed for physical neglect from the Modified Multidimensional Neglectful Behavior Scale (MNBS-A; Straus et al., 1995); Phys. Neg. Frequency = Frequency of physical neglect assessed through the MNBS-A; Emo. Neg. Quality = Number of items endorsed for emotional neglect from the MNBS-A; Emo. Neg. Frequency = Frequency of physical neglect assessed via the MNBS-A; Phys. Abuse Quality = Number of items endorsed for physical abuse using a project-developed self-report measure of maltreatment; Phys. Abuse Frequency = Frequency of physical abuse reported; Emo. Abuse Quality = Number of items endorsed for emotional abuse; Emo. Abuse Frequency = Frequency of emotional abuse reported; Sex. Abuse Quality = Number of items endorsed for sexual abuse; Sex. Abuse Frequency = Frequency of sexual abuse; Age 12 Psychopathology = Diagnostic status reflecting clinically significant levels of depression, anxiety, anger, post-traumatic stress, or dissociation at Baseline on the Trauma Symptom Checklist for Children (Briere, 1996). Age 16 Psychopathology = Diagnostic status reflecting clinically significant levels of depression, anxiety, anger, post-traumatic stress, or dissociation at age 16 on the Trauma Symptom Checklist for Children (Briere, 1996); Age 18 Psychopathology = Diagnostic status reflecting clinically significant levels of depression, anxious arousal, anger/irritability, intrusive experiences, defensive avoidant, or dissociation at age 18 on the Trauma Symptom Inventory (Briere, 1995). Age 12, 16, and 18 psychopathology values denote the number of adolescents endorsing distress at each age, while values in the parentheses indicate the percentages of adolescents meeting threshold within each subpopulation. For gender, t-scores compared males and females. For race, t-scores compared those who identified with a given race compared to who did not. All significant differences reflect elevated levels of the measure.

*p <.05; **p <.01

As for missing data, 19.2% of the sample had missing data at age 16, 10.6% of the sample had missing data at age 18, and 14.3% had missing data at both ages 16 and 18. Relatedly, Little’s MCAR test was significant, χ^2^(59) = 148.65; *p* <.01, suggesting the data were not missing completely at random. Subsequently, an independent samples *t*-test was conducted to examine if “completers” differed from someone who had missing follow-up data. Additionally, regression analyses were conducted to examine whether the relationship between indices of maltreatment and psychological distress at each age differed as a function of missingness. Overall, results from the *t-*tests were non-significant, *p*s > 0.05, with the exception of emotional neglect, *t*= −2.09; *p* =.04, such that those who reported elevated emotional neglect at baseline were more likely to miss a follow-up assessment. Similarly, results from the regression analyses revealed no significant effects of missingness on the relationship between maltreatment and psychopathology at each wave, *p*s > 0.05, with the exception of sexual abuse frequency and psychopathology at age 18, *B* = 0.13; *SE* = 0.06; *p* <.05. Thus, any significant findings concerning emotional neglect and sexual abuse frequency would require subsequent analyses to ensure they are not biased to this result. However, as the overwhelming pattern of findings suggested data was missing at random (MAR), the expectation maximization (EM) algorithm utilizing continuous, raw scores of maltreatment exposure, TSCC, and TSI scores, as well as adolescent gender and race/ethnicity was used to impute the missing data as recommended (Schafer & Graham, 2002).

Finally, we examined which maltreatment experiences and trauma-related psychopathology were significant and robust to demographic characteristics (i.e., gender, race/ethnicity, recruitment site) in a series of logistic regression models. At age 12, results demonstrated that both quality and frequency indicators of cumulative risk, emotional neglect, physical abuse, and emotional abuse, as well as sexual abuse quality (but not frequency) were significantly associated with trauma-related mental health disorders, *p*s < 0.05. As for prospective outcomes controlling for age 12 psychopathology, we found that both indicators (i.e., quality and frequency) of cumulative risk, physical abuse, emotional abuse, and sexual abuse, along with physical neglect quality (but not frequency), were significantly associated with distress at age 16, *p*s < 0.05. As for age 18, cumulative frequency and physical abuse frequency (but not quality), both indicators (i.e., quality and frequency) of emotional abuse and sexual abuse demonstrated a significant association with psychopathology. Thus, subsequent tests of accuracy and fairness focused only on significant indices of maltreatment-exposure identified at each age. Results for the logistic regression models are presented in Appendix C.

### Accuracy

Discrimination and calibration statistics are presented in Table 2. Regarding age 12 trauma-related mental health disorders, examination of AUCs revealed both conceptualizations of cumulative maltreatment exposure (i.e., quality and frequency) to be equivalent, AUCs = 0.69, achieving at least a significant medium effect size (i.e., AUC ≥ 0.64). Next, we examined differences across, and within, maltreatment subtypes. Overall, we found that only exposure to emotional abuse, both the quality and frequency, demonstrated AUCs in the clinically useful range, AUCs *≥* 0.70. Relatedly, examination of DeLong’s tests revealed AUCs of emotional abuse to be similar to AUCs for cumulative risk, *ps >* 0.05, but significantly greater than AUCs of other maltreatment subtypes, *ps <* 0.05.^1^ In contrast, for prospective outcomes, only cumulative frequency, AUC = 0.64, and emotional abuse frequency, AUC = 0.64, demonstrated accuracy in predicting outcomes at age 16, while no maltreatment subtype was found to demonstrate accurate levels of discrimination in classifying psychological distress at age 18, AUCs < 0.64.

**Table 2:** Discrimination and calibration statistics in the full sample

Indices	AUC	Intercept (CIL)	Slope	Emax	Spiegelhalter’s z	Spiegelhalter’s *p*
Age 12 Psychopathology (*n* = 41)						
Cumulative Quality	0.69** (0.61,** 0.78)**	0 (−1.26, 1.17)	1 (0.58, 1.42)	0.06 (0.02, 0.19)	0.02	0.98
Cumulative Frequency	0.69** (0.61,** 0.78)**	0 (−1.33, 1.19)	1 (0.56, 1.41)	0.17 (0.07, 0.29)	0.11	0.91
Emo. Neg. Quality	0.58 (0.48, 0.67)	0 (−3.45, 2.21)	1 (−0.12, 1.76)	0.03 (0.02, 0.18)	0	1
Emo. Neg. Frequency	0.60* (0.50, 0.69)	0 (−3.67, 2.39)	1 (−0.20, 1.83)	0.05 (0.05, 0.21)	0.01	0.99
Phys. Abuse Quality	0.62* (0.52, 0.71)	0 (−2.30, 1.76)	1 (0.26, 1.61)	0.10 (0.04, 0.20)	0.03	0.98
Phys. Abuse Frequency	0.61* (0.51, 0.71)	0 (−1.75, 1.36)	1 (0.43, 1.45)	0.13 (0.06, 0.32)	0.02	0.98
Emo. Abuse Quality	0.70** (0.61,** 0.79)**	0 (−1.19, 1.04)	1 (0.62, 1.39)	0.05 (0.03, 0.20)	0.05	0.96
Emo. Abuse Frequency	0.70** (0.61,** 0.79)**	0 (−1.11, 1.07)	1 (0.64, 1.36)	0.11 (0.06, 0.33)	0.12	0.91
Sex. Abuse Quality	0.61* (0.51, 0.70)	0 (−2.75, 1.93)	1 (0.11, 1.67)	0.09 (0.05, 0.20)	0.02	0.98
Age 16 Psychopathology (*n* = 37)						
Cumulative Quality	0.61* (0.51, 0.70)	0 (−2.77, 2.48)	1 (0.12, 1.84)	0.02 (0.01, 0.15)	0	1
Cumulative Frequency	0.64** (0.54,** 0.73)**	0 (−2.30, 1.61)	1 (0.29, 1.55)	0.07 (0.05, 0.22)	0.03	0.98
Phys. Neg. Quality	0.55 (0.45, 0.65)	0 (−3.67, 2.01)	1 (−0.15, 1.69)	0.01 (0.02, 0.27)	0	1
Phys. Abuse Quality	0.58 (0.45, 0.65)	0 (−3.03, 2.08)	1 (0.05, 1.70)	0.05 (0.02, 0.18)	0.01	0.99
Phys. Abuse Frequency	0.58 (0.48, 0.69)	0 (−2.69, 1.76)	1 (0.14, 1.60)	0.10 (0.04, 0.22)	0.02	0.98
Emo. Abuse Quality	0.63** (0.54, 0.73)	0 (−2.31, 1.70)	1 (0.27, 1.58)	0.03 (0.03, 0.16)	0.01	0.99
Emo. Abuse Frequency	0.65** (0.55,** 0.75)**	0 (−2.24, 1.72)	1 (0.30, 1.58)	0.10 (0.09, 0.19)	0.03	0.98
Sex. Abuse Quality	0.55 (0.45, 0.65)	0 (−3.57, 1.86)	1 (−0.11, 1.65)	0.11 (0.11, 0.33)	− 0.03	0.98
Sex. Abuse Frequency	0.55 (0.45, 0.65)	0 (−3.56, 1.84)	1 (−0.10, 1.62)	0.06 (0.05, 0.37)	− 0.02	0.99
Age 18 Psychopathology (*n* = 121)						
Cumulative Frequency	0.57* (0.52, 0.63)	0 (−1.52, 1.52)	1 (0.16, 1.84)	0.14 (0.07, 0.29)	0.02	0.98
Phys. Abuse Frequency	0.56* (0.50, 0.62)	0 (−1.40, 1.40)	1 (0.22, 1.78)	0.11 (0.06, 0.30)	0.02	0.99
Emo. Abuse Quality	0.56* (0.50, 0.62)	0 (−1.32, 1.22)	1 (0.29, 1.67)	0.09 (0.05, 0.21)	0.02	0.99
Emo. Abuse Frequency	0.57* (0.51, 0.62)	0 (−1.38, 1.29)	1 (0.25, 1.72)	0.11 (0.06, 0.28)	0.02	0.98
Sex. Abuse Quality	0.55 (0.49, 0.61)	0 (−1.15, 0.95)	1 (0.38, 1.55)	0.02 (0.04, 0.26)	0	1
Sex. Abuse Frequency	0.55 (0.49, 0.61)	0 (−1.34, 1.29)	1 (0.27, 1.73)	0.12 (0.10, 0.40)	0.02	0.98

*Note*: Bold = AUCs above 0.64 threshold; CIL = Calibration-in-the-Large; Cumulative Quality = Total number of maltreatment subtypes endorsed at Baseline (Age 12); Cumulative Frequency = Sum of the frequency of each subtype divided by the number of items used to assess each subtype, respectively, endorsed at Baseline; Emo. Neg. Frequency = Frequency of physical neglect assessed via the MNBS-A; Phys. Abuse Quality = Number of items endorsed for physical abuse using a project-developed self-report measure of maltreatment; Phys. Abuse Frequency = Frequency of physical abuse reported; Emo. Abuse Quality = Number of items endorsed for emotional abuse; Emo. Abuse Frequency = Frequency of emotional abuse reported; Sex. Abuse Quality = Number of items endorsed for sexual abuse; Sex. Abuse Frequency = Frequency of sexual abuse. Age 12 Psychopathology = Diagnostic status reflecting clinically significant levels of depression, anxiety, anger, post-traumatic stress, or dissociation at Baseline on the Trauma Symptom Checklist for Children (Briere, 1996). Age 16 Psychopathology = Diagnostic status reflecting clinically significant levels of depression, anxiety, anger, post-traumatic stress, or dissociation at age 16 on the Trauma Symptom Checklist for Children (Briere, 1996); Age 18 Psychopathology = Diagnostic status reflecting clinically significant levels of depression, anxious arousal, anger/irritability, intrusive experiences, defensive avoidant, or dissociation at age 18 on the Trauma Symptom Inventory (Briere, 1995)

*p <.05, **p <.01

Next, we tested accuracy from a calibration perspective. Overall, results revealed that cumulative risk models defined by maltreatment quality and frequency were well-calibrated for predicting mental health disorders across all three ages (i.e., Spiegelhalter’s *ps* < 0.05; confidence intervals for CIL including 0; confidence intervals for slopes including 1). Relatedly, Emax scores between the two indices suggested similar levels of calibration performance between the two approaches. Regarding maltreatment subtypes, results revealed all subtypes to be well calibrated in predicting concurrent and prospective diagnoses. Emax scores also suggested no significant differences across subtypes. Given these results, tests of fairness focused on indices of maltreatment demonstrating accurate discrimination and calibration at each age. Specifically, at age 12 we tested the fairness of cumulative quality and frequency, and emotional abuse quality and frequency. At age 16 we tested the fairness of cumulative frequency and emotional abuse frequency; however, as no indices were accurate for age 18 outcomes, these outcomes were not further tested.

### Fairness

We first examined patterns of potential unfairness with regard to statistical discrimination. For gender, AUCs were similar across indices of maltreatment at each age, *p*s > 0.05. Meanwhile, patterns of unfairness emerged across race/ethnicity, with cumulative maltreatment frequency demonstrating significantly greater accuracy in predicting age 12 psychopathology among youth identifying as White, AUC = 0.84, compared to those identifying as Black, AUC = 0.67, *p* <.05. For prospective psychopathology, DeLong’s tests indicated no significant differences in AUCs across race/ethnicity, *p*s > 0.05.

**Fig. 1:**
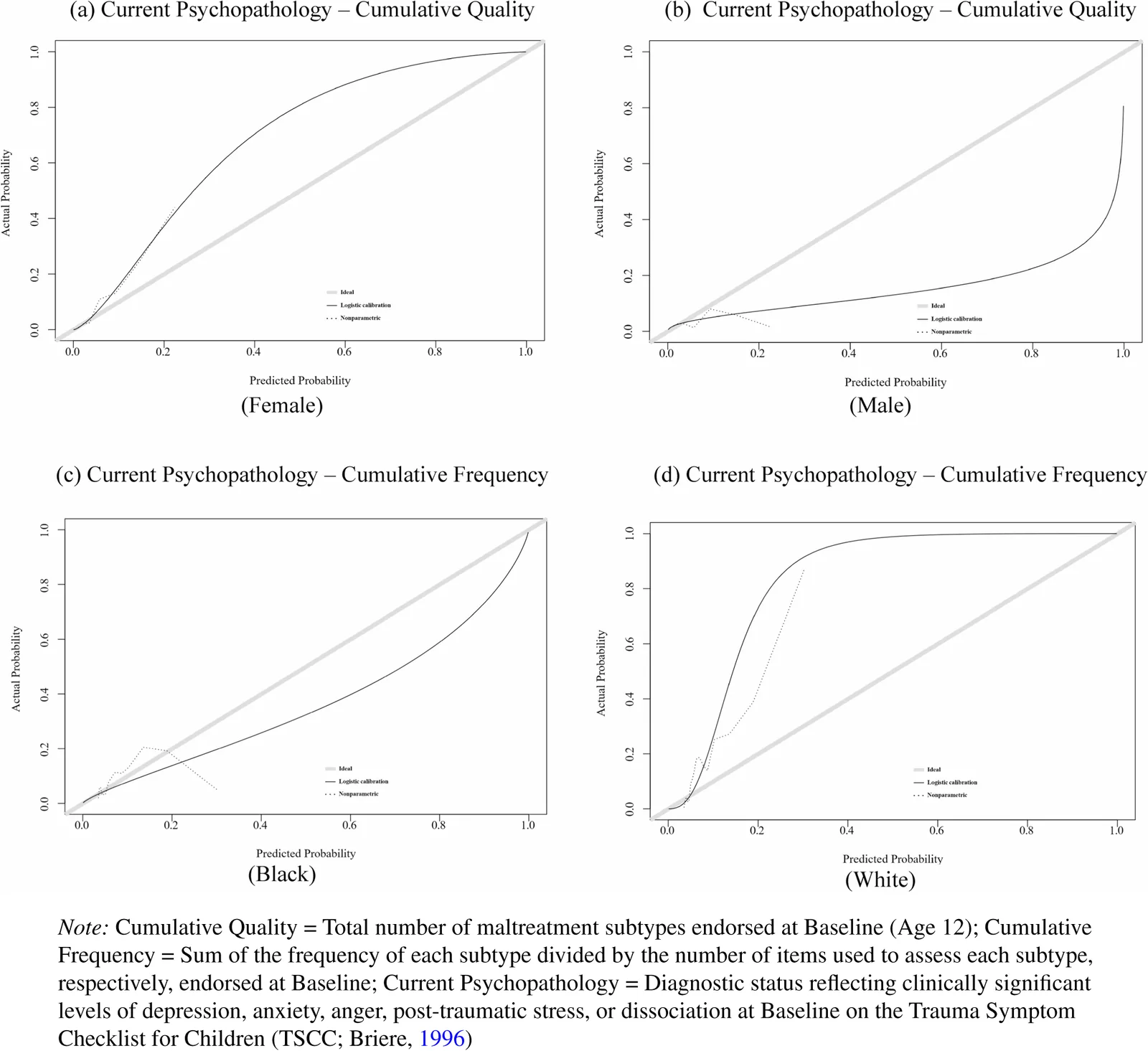
Calibration Curves Depicting Patterns of Unfairness across Gender and Race/Ethnicity

With regard to calibration, patterns of unfairness emerged across gender when predicting risk for psychopathology at age 12. Specifically, cumulative maltreatment quality was found to overestimate concurrent trauma-related psychopathology among male (i.e., confidence interval for CIL < 0), but not female adolescents. As for race/ethnicity, results for cumulative frequency models suggested patterns of underestimation (i.e., confidence interval for CIL > 0), as well as overly modest estimation of concurrent risk among adolescents identifying as White (i.e., confidence interval for slope > 1), but not as Black. Calibration curves depicting patterns of unfairness within concurrent cumulative risk models are presented in Fig. 1. Of particular relevance, within each subpopulation, no pattern of miscalibration nor unfairness emerged for emotional abuse (Table 3) in predicting concurrent psychopathology. These findings suggest that emotional abuse, as measured by both quality and frequency, provides accurate and equitable risk predictions for concurrent trauma-related mental health outcomes in adolescents. For age 16, results for both cumulative and emotional abuse frequency were inconclusive due to impermissible solutions (e.g., 95% confidence intervals did not include the observed values). Thus, while both cumulative and emotional abuse frequency demonstrated statistical fairness for prospective outcomes regarding discrimination, results concerning calibration were inconclusive.

**Table 3:** Discrimination and calibration statistics for cumulative and emotional abuse indices across adolescent subgroups

Indices	AUC	Intercept (CIL)	Slope	Emax	S’z	S’*p*	AUC	Intercept (CIL)	Slope	Emax	S’z	S’*p*
	Female						Male					
Age 12 Psychopathology (*n* = 14 [Male]; *n* = 27 [Female])												
Cumulative Quality	0.75** (0.65,** 0.85)**	1.44 (−0.22, 3.18)	1.41 (0.85, 2.09)	0.22 (0.05, 0.55)	1.37	0.17	0.60 (0.43, 0.86)	−1.90 (−4.65, − 0.04)	0.48 (−0.35, 1.12)	0.21 (0.13, 0.25)	−1.35	0.18
Cumulative Frequency	0.76** (0.66,** 0.86)**	1.13 (−0.56, 3.28)	1.29 (0.73, 1.99)	0.15 (0.10, 0.44)	1.47	0.14	0.58 (0.42, 0.75)	−1.42 (−6.95, 0.63)	0.65 (−1.11, 1.37)	0.24 (0.14, 0.31)	−1.33	0.18
Emo. Abuse Quality	0.74** (0.64,** 0.84)**	0.30 (−1.12, 1.64)	1.02 (0.54, 1.49)	0.08 (0.05, 0.27)	1.26	0.21	0.60 (0.42, 0.78)	− 0.58 (−4.33, 1.24)	0.92 (−0.22, 1.64)	0.06 (0.06, 0.26)	−1.23	0.22
Emo. Abuse Frequency	0.74** (0.64,** 84)**	− 0.11 (−1.35, 1.58)	0.86 (0.48, 1.44)	0.21 (0.11, 0.38)	1.48	0.14	0.61 (0.44, 0.79)	− 0.16 (−3.31, 1.49)	1.07 (0.05, 1.71)	0.06 (0.06, 0.38)	−1.36	0.17
Age 16 Psychopathology (*n* = 16 [Male]; *n* = 21 [Female])												
Cumulative Frequency	0.67* (0.54,** 0.79)**	0.73 (−2.43, 3.05)	1.20 (0.20, 1.97)	0.06 (0.06, 0.43)	0.57	0.57	0.61 (0.47, 0.74)	− 0.77 (−7.38, 1.72)	0.79 (−1.20, 1.67)	0.14 (0.09, 0.28)	− 0.54	0.59
Emo. Abuse Frequency	0.67** (0.55,** 0.80)**	0.16 (−2.60, 2.45)	1.02 (0.12, 1.78)	0.11 (0.09, 0.27)	0.48	0.63	0.62 (0.47, 0.77)	− 0.28 (−5.93, 2.53)	0.95 (−0.81, 1.89)	0.10 (0.07, 0.23)	− 0.46	0.65
	Black						White					
Age 12 Psychopathology (*n* = 20 [Black]; *n* = 11 [White])												
Cumulative Quality	0.68** (0.55,** 0.81)**	− 0.33 (−2.35, 1.41)	0.92 (0.31, 1.61)	0.12 (0.04, 0.23)	− 0.46	0.64	0.79** (0.69,** 0.90)**	1.58 (−0.72, 4.14)	1.58 (0.84, 2.73)	0.16 (0.04, 0.56)	− 0.03	0.98
Cumulative Frequency	0.67* (0.54,** 0.80)**	− 0.74 (−2.96, 0.89)	0.79 (0.10, 1.34)	0.26 (0.18, 0.31)	− 0.44	0.66	0.84** (0.74,** 0.95)**	4.47 (0.97, 10.19)	2.51 (1.36, 4.53)	0.57 (0.10, 0.79)	0.11	0.91
Emo. Abuse Quality	0.67** (0.55,** 0.80)**	− 0.59 (−2.63, 0.91)	0.83 (0.17, 1.34)	0.13 (0.05, 0.27)	− 0.27	0.79	0.84** (0.72,** 0.96)**	1.78 (−0.40, 4.01)	1.68 (0.94, 2.70)	0.24 (0.07, 0.69)	− 0.16	0.87
Emo. Abuse Frequency	0.67** (0.54,** 0.80)**	− 0.40 (−2.70, 1.07)	0.90 (0.20, 1.41)	0.12 (0.08, 0.35)	− 0.40	0.69	0.85** (0.73,** 0.97)**	2.41 (−0.37, 7.00)	1.83 (0.95, 3.41)	0.23 (0.13, 0.69)	0.12	0.90
Age 16 Psychopathology (*n* = 18 [Black]; *n* = 13 [White])												
Cumulative Frequency	0.65* (0.52,** 0.78)**	0.09 (−3.24, 2.32)	1.08 (0.02, 1.82)	0.18 (0.11, 0.38)	− 0.59	0.58	0.63 (0.46, 0.81)	.64^a^	1.09^a^	1.09^a^	1.31	0.19
Emo. Abuse Frequency	0.66* (0.52,** 0.80)**	0.63 (−3.00, 2.88)	1.25 (0.10, 2.04)	.02^a^	− 0.49	0.63	0.60 (0.43, 0.78)	−.10^a^	.86^a^	.23^a^	1.18	0.24

*Note*: See Table 1 for information on individual indices and outcomes. Bold = AUCs above 0.64 threshold; CIL = Calibration-in-the-Large; S’z = Spiegelhalter’s Z; S’p = Spiegelhalter’s p.

^a^Results demonstrated impermissible solutions (e.g., 95% Cis did not include the observed value).

*p <.05, **p <.01.

## Discussion

The present study sought to inform emerging adversity screening protocols. Overall, we found equivocal findings when conceptualizing patterns of maltreatment by quality versus frequency; however, notable patterns of *statistical unfairness* emerged across maltreatment conceptualizations. Specifically, results demonstrated cumulative quality to overestimate risk for psychopathology among males, but not females when predicting concurrent outcomes. Across race/ethnic subgroups, results showed greater accuracy of cumulative frequency predicting concurrent outcomes among youth identifying as White, as well as patterns of underestimation and overly modest predictions of risk when compared to their peers who identified as Black. Meanwhile, examination of accuracy and fairness found that emotional abuse may be more important to prioritize in universal screening initiatives for adolescents. Results at age 16 found evidence suggesting cumulative frequency and emotional abuse frequency to provide accurate and potentially equitable estimates of short-term prospective distress, while all maltreatment indices failed to demonstrate clinical utility in predicting long-term (i.e., age 18) prospective psychopathological outcomes. Below, we discuss both the research and clinical implications of our study.

To date, most screening protocols define adversity-exposure based on quality (i.e., number of different adversity subtypes experienced). More recent conceptualizations have further suggested that this approach should specifically focus on maltreatment given that it may be more closely tethered to behavioral well-being relative to other commonly assessed adversities (e.g., impaired caregiver due to substance use; Negriff, 2020). Yet, these approaches still focused on subtypes, and did not account for the incremental validity of indexing maltreatment via frequency (i.e., how many times one was exposed to experiences of maltreatment). Given findings from population health research (e.g., Vachon et al., 2015; Ellenbogen et al., 2013), we predicted prioritizing frequency of maltreatment, regardless of subtype, would lead to more accurate results. It is important to note that cumulative maltreatment quality and frequency results demonstrated comparable levels of accuracy across both statistical discrimination and calibration to previous research examining types of trauma exposure beyond maltreatment (e.g., ACEs; Cohen & Choi, 2022). While these results are incongruent with our original hypotheses, they suggest little is lost (or gained) when classifying concurrent risk focused on maltreatment experiences specifically relative to ACEs broadly. Interestingly, while findings demonstrated similar results in the clinical accuracy of screening for cumulative maltreatment exposure quality when compared to frequency in predicting concurrent risk, potential differences emerged when predicting short-term (i.e., age 16) prospective risk. These findings potentially suggest that more accurate forecasts of adolescent mental health are possible when focusing on the number of instances, rather than the number of types, of maltreatment exposure.

Our study’s focus on statistical fairness further clarifies some potential pitfalls when screening for environmental risk in applied settings. Examination of discrepancies in statistical discrimination and calibration across adolescent subpopulations demonstrated patterns of unfairness across both gender and race when predicting concurrent outcomes. Specifically, polyvictimization based on subtypes and frequency overestimated the likelihood of risk for trauma-related psychopathology in male adolescents, while relying on frequency of maltreatment led to patterns of underestimation and overly modest predictions of risk in White adolescents. These findings, particularly regarding race/ethnicity, resemble results of previous population health studies which have similarly demonstrated patterns of unfairness across adolescent subpopulations when assessing adversities more broadly (e.g., ACEs; Cohen & Choi, 2022). Translationally, patterns of statistical unfairness with regard to calibration may be particularly problematic, because it undermines the use of assessing maltreatment exposure for public purposes as advocated for by proponents of ACEs screening (Anda et al., 2020).

These patterns of unfairness across adolescent subpopulations may be attributable to multiple explanations. For example, the unfairness in assessment of risk across gender may be related to girls having depleted resources in adolescence due to increased exposure to interpersonal adversities (e.g., Alisic et al., 2014; Salk et al., 2017). These differences may be due to sociocultural factors, including socially influenced gender differences in response to distress, differences in cognitive responses to adversity exposure (e.g., greater self-blame among female adolescents), and differences in behavioral coping strategies (e, g., greater utilization of wishful thinking, mental disengagement, and suppression of adversity memories among females; see Tolin & Foa, 2008), as well as, neurobiological differences that may be particularly important for adolescents undergoing puberty (Garza & Jovanovic, 2017). Relatedly, the patterns of unfairness across race/ethnicity congruent with previous research (e.g., Cohen & Choi, 2022) may also be due to multiple explanations. For example, in line with resilience theories, research demonstrates that factors such as religiosity and spirituality, support from religious organizations, and communalism may be especially helpful for adolescents who identify as Black in protecting against negative outcomes of adversity exposure (Woods-Jaeger et al., 2021), leading to differences in the relationship between adversity and psychopathology across racially diverse youth. Alternatively, in line with a *steeling hypothesis*, non-White adolescents tend to experience more adversity due to greater exposure to poverty, and as a result may be more resilient to maltreatment-related outcomes (Hunt et al., 2017).

Instead of relying on cumulative maltreatment indices for classifying risk, our findings suggest it may be more helpful to focus on adversities that rely on verbal communication within the family. Specifically, our current study found that exposure to emotional abuse, both quality and frequency, was particularly important in classifying concurrent and short-term prospective risk across adolescent gender and race/ethnic subgroups. These findings are in line with recent basic research which demonstrated that emotional abuse may be particularly harmful for gender and racially/ethnically diverse adolescents through its impact on both intrapersonal mechanisms, including emotion regulation and negative cognitive biases (Burns et al., 2010), physiological stress response systems (Duprey et al., 2021), and neurobiology (Heim et al., 2013), as well as interpersonal mechanisms, such as decreases in the availability of social support (Wang et al., 2021; Cecil et al., 2017). Relatedly, previous research has demonstrated exposure to emotional abuse, measured through both quality and frequency, to be associated with the highest incidence rates of revictimization and distress severity when compared to other maltreatment subtypes (Gama et al., 2021), which may explain the similarity in findings observed across quality and frequency of emotional abuse. That emotional abuse conferred greater classification risk for psychopathology relative to other maltreatment experiences further emphasizes the need to consider alternative approaches to operationalizing early adversities beyond cumulative scores (e.g., ACEs; Negriff, 2020), such as differentially weighting certain maltreatment experiences (Lacey & Minnis, 2020).

Finally, it is important to note that none of our findings suggested that relying on maltreatment-exposure to forecast long-term future mental health problems. Our findings replicate past research which found ACEs scores between birth and age 12 demonstrated discriminatory capacity just above chance (AUC = 0.58) when predicting prospective mental health outcomes at age 18 (Baldwin et al., 2021). Although others have suggested that focusing on maltreatment experiences may improve the accuracy of ACEs screening (Negriff, 2020), we did not find support for this claim. These results bring into question the justification of long-term benefits for emerging screening practices. For example, in the state of California, statewide policies have been put in place to promote universal screening for exposure to early adversities. Such policies include reimbursing Medicaid (Medi-Cal in California) service providers $29 per individual screened for ACEs and mandating commercial insurance coverage of ACEs screening (ACEs Aware; Shimkhada et al., 2022), aimed at identifying individuals who may be at-risk for negative health outcomes using ACEs scores measured either retrospectively or prospectively (Purewal et al., 2016; Lacey & Minnis, 2020). Importantly, however, findings from the current study, as well as others (e.g., Baldwin et al., 2021) suggest relying on adversity assessments as currently constituted may lead to more potential harm (e.g., stigma, inappropriate referrals; see Finkelhor, 2018 for a discussion) than public health benefit. Alternatively, more recent studies (e.g., Cohen et al., 2025a, b) have suggested that incorporating assessments of *dynamic risks* (i.e., modifiable individual differences stemming from adversity exposure) may be more predictive of mental health outcomes and more feasible to implement. Thus, while retrospective assessments of adversities in adults have laid the foundation for pediatric settings screening youth for adversities (Shimkhada et al., 2022), the reasoning for why it is justified to ask these sensitive questions needs to be updated based on longitudinal studies, including this one, with youth.

Despite the methodological strengths of the current study, several limitations should be noted. First, the study was unable to investigate patterns of statistical unfairness across more diverse race/ethnic adolescent subgroups (e.g., Hispanic adolescents) due to limitations in sample size. Given previous findings demonstrating significant biases in statistical fairness among Hispanic adolescents, as well as adolescent subpopulations based on intersecting race/ethnicity and gender (Cohen & Choi, 2022), it may be important for future studies to examine statistical fairness across diverse adolescent subgroups. Relatedly, patterns of impermissible solutions that emerged regarding calibration statistics, particularly within adolescent subpopulations and specific maltreatment subtypes (e.g., emotional abuse frequency at age 16), may be attributable to limitations in sample size (e.g., Caye et al., 2022; Cunha et al., 2023). Thus, replication of the current study in a larger sample may be necessary to show statistical discrimination *and* calibration fairness for short-term prospective outcomes using cumulative and emotional abuse frequency as screening indices. Next, while the current study focused on maltreatment quality and frequency, it may be important to test the clinical utility of alternative indices of maltreatment that are able to integrate across multiple dimensions (e.g., chronicity and timing; Russotti et al., 2021), as they may provide more comprehensive assessments of maltreatment exposure. Additionally, although the utilization of a large secondary dataset allowed for the examination of accuracy and fairness across adolescent subgroups, the current study was simultaneously constrained by the coding schemes provided in the dataset in which disparate experiences of maltreatment within each subtype were equated under a unitary code (e.g., sexual abuse). Investigating more fine-grained approaches of measuring and indexing maltreatment exposure may help better understand the clinical utility of screening for different dimensions of maltreatment. Finally, maltreatment exposure and mental health outcomes relied on adolescent retrospective self-reports. While relying on adolescent self-reports may allow for a “child-focused” examination of maltreatment (Proctor & Dubowitz, 2014), a multi-informant method may allow for a more comprehensive examination of trauma exposure and related functioning (De Los Reyes et al., 2015; Cohen & Thakur, 2021), while also reducing the risk of retrospective recall bias.

Overall, the present study found similar results across quality and frequency dimensions of maltreatment when assessing concurrent mental health risk, while demonstrating potential differences when predicting short-term prospective outcomes. Importantly, we found that focusing on emotional abuse to index adversity risk may be a more promising approach. From a methodological perspective, our results highlighted the importance of examining statistical fairness in trauma screening alongside more traditional tests of accuracy (Youngstrom, 2014), which may help prevent potential biases from emerging in universal trauma screening protocols. Translationally, these findings echo previous concerns regarding the implementation of, and reliance on, current universal trauma-screening initiatives (e.g., ACEs; Finkelhor, 2018; Baldwin et al., 2021; Cohen & Choi, 2022). Further, results from the current study expand on this line of work by suggesting that differentially weighing specific adversities (e.g., emotional abuse) may be an alternative approach to conventional trauma screening practices that may provide a more accurate and equitable process for identifying risk among adolescents.

## Supplementary Information

Below is the link to the electronic supplementary material.

Supplementary Material 1

## Data Availability

The Longitudinal Studies on Child Abuse and Neglect (LONGSCAN) data is available through the National Data Archive on Child Abuse and Neglect (NDACAN).

## Footnotes

1 Of note, results from DeLong’s tests indicated non-significant difference in AUCs between quality of emotional abuse and both physical (*z* = 1.87; *p* =.06) and sexual abuse (*z* = 1.58; *p* =.11). However, further analyses examining results of DeLong’s tests between cumulative risk and physical and sexual abuse demonstrated significant differences between the AUCs (*p*s < 0.05). Thus, it was determined that emotional and the cumulative risk models conferred the greatest risk.
