# Supplementary material for: Maltreatment-Exposure Screening and Adolescent Mental Health: An Evidence-Based Medicine Perspective on Accuracy and Fairness
Source: J Child Adolesc Trauma. 2025 Nov 8;19(3):995–1009. doi: 10.1007/s40653-025-00767-1 (PMC13546273; doi:10.1007/s40653-025-00767-1)
Supplement: Supplementary file 1 [file 40653_2025_767_MOESM1_ESM.docx]

Appendix A. Maltreatment Variables Recoding Scheme

| Variable | Original Value | Original Number of Cases | Recoded Value | Recoded Number of Cases |
| --- | --- | --- | --- | --- |
| Physical Neglect Quality | 0 | 751 | 0 | 751 |
|  | 1 | 61 | 1 | 61 |
|  | 2 | 12 | 2 | 12 |
|  | 3 | 5 | 4 | 15 |
|  | 4 | 5 |  |  |
|  | 6 | 1 |  |  |
|  | 7 | 4 |  |  |
| Physical Neglect Frequency | 0 | 768 | 0 | 768 |
|  | 1 | 34 | 1 | 34 |
|  | 2 | 25 | 2 | 25 |
|  | 3 | 1 | 5.5 | 12 |
|  | 4 | 3 |  |  |
|  | 5 | 2 |  |  |
|  | 6 | 2 |  |  |
|  | 14 | 4 |  |  |
| Emotional Neglect Quality | 0 | 562 | 0 | 570 |
|  | 1 | 167 | 1 | 171 |
|  | 2 | 46 | 2 | 47 |
|  | 3 | 31 | 3 | 35 |
|  | 4 | 14 | 4 | 15 |
|  | 5 | 5 | 6 | 19 |
|  | 6 | 5 |  |  |
|  | 7 | 9 |  |  |
| Emotional Neglect Frequency | 0 | 608 | 0 | 623 |
|  | 1 | 78 | 1 | 78 |
|  | 2 | 78 | 2 | 80 |
|  | 3 | 18 | 3 | 18 |
|  | 4 | 18 | 4 | 19 |
|  | 5 | 7 | 5 | 13 |
|  | 6 | 6 |  |  |
|  | 7 | 4 | 8 | 12 |
|  | 8 | 8 |  |  |
|  | 9 | 2 | 12 | 14 |
|  | 10 | 3 |  |  |
|  | 12 | 4 |  |  |
|  | 13 | 1 |  |  |
|  | 14 | 4 |  |  |
| Physical Abuse Quality | 0 | 651 | 0 | 651 |
|  | 1 | 115 | 1 | 115 |
|  | 2 | 32 | 2 | 32 |
|  | 3 | 21 | 3 | 21 |
|  | 4 | 10 | 4 | 10 |
|  | 5 | 6 | 5 | 10 |
|  | 7 | 2 |  |  |
|  | 9 | 1 |  |  |
|  | 10 | 1 |  |  |
| Physical Abuse Frequency | 0 | 688 | 0 | 688 |
|  | 1 | 48 | 1 | 48 |
|  | 2 | 37 | 2 | 37 |
|  | 3 | 16 | 3 | 16 |
|  | 4 | 12 | 4 | 12 |
|  | 5 | 5 | 6 | 12 |
|  | 6 | 7 |  |  |
|  | 7 | 7 | 8 | 16 |
|  | 8 | 9 |  |  |
|  | 9 | 1 | 10.5 | 10 |
|  | 10 | 4 |  |  |
|  | 11 | 1 |  |  |
|  | 14 | 1 |  |  |
|  | 18 | 1 |  |  |
|  | 21 | 2 |  |  |
| Emotional Abuse Quality | 0 | 466 | 0 | 466 |
|  | 1 | 132 | 1 | 132 |
|  | 2 | 69 | 2 | 69 |
|  | 3 | 44 | 3 | 44 |
|  | 4 | 39 | 4 | 39 |
|  | 5 | 21 | 5 | 21 |
|  | 6 | 17 | 6 | 17 |
|  | 7 | 19 | 7 | 19 |
|  | 8 | 13 | 8 | 13 |
|  | 9 | 6 | 10 | 19 |
|  | 10 | 5 |  |  |
|  | 11 | 4 |  |  |
|  | 12 | 1 |  |  |
|  | 13 | 1 |  |  |
|  | 14 | 1 |  |  |
|  | 15 | 1 |  |  |
| Emotional Abuse Frequency | 0 | 519 | 0 | 519 |
|  | 1 | 71 | 1 | 71 |
|  | 2 | 62 | 2 | 62 |
|  | 3 | 36 | 3 | 36 |
|  | 4 | 32 | 4 | 32 |
|  | 5 | 18 | 5 | 18 |
|  | 6 | 23 | 6 | 23 |
|  | 7 | 15 | 7 | 15 |
|  | 8 | 11 | 8 | 11 |
|  | 9 | 7 | 9 | 12 |
|  | 10 | 5 |  |  |
|  | 11 | 4 | 12 | 10 |
|  | 12 | 6 |  |  |
|  | 13 | 4 | 16 | 13 |
|  | 15 | 1 |  |  |
|  | 16 | 3 |  |  |
|  | 17 | 5 |  |  |
|  | 18 | 3 | 24 | 17 |
|  | 19 | 2 |  |  |
|  | 20 | 1 |  |  |
|  | 22 | 2 |  |  |
|  | 24 | 2 |  |  |
|  | 26 | 1 |  |  |
|  | 29 | 2 |  |  |
|  | 36 | 1 |  |  |
|  | 40 | 1 |  |  |
|  | 41 | 1 |  |  |
|  | 57 | 1 |  |  |
| Sexual Abuse Quality | 0 | 709 | 0 | 709 |
|  | 1 | 56 | 1 | 56 |
|  | 2 | 20 | 2 | 20 |
|  | 3 | 21 | 3 | 21 |
|  | 4 | 11 | 4 | 16 |
|  | 5 | 5 |  |  |
|  | 6 | 10 | 6 | 17 |
|  | 7 | 3 |  |  |
|  | 8 | 3 |  |  |
|  | 9 | 1 |  |  |
| Sexual Abuse Frequency | 0 | 733 | 0 | 733 |
|  | 1 | 20 | 1 | 20 |
|  | 2 | 21 | 2 | 21 |
|  | 3 | 8 | 4 | 17 |
|  | 4 | 9 |  |  |
|  | 5 | 8 | 6 | 18 |
|  | 6 | 10 |  |  |
|  | 7 | 3 | 8 | 10 |
|  | 8 | 6 |  |  |
|  | 9 | 1 |  |  |
|  | 10 | 1 | 12 | 10 |
|  | 11 | 2 |  |  |
|  | 12 | 3 |  |  |
|  | 13 | 1 |  |  |
|  | 14 | 3 |  |  |
|  | 16 | 2 | 19 | 10 |
|  | 17 | 2 |  |  |
|  | 19 | 2 |  |  |
|  | 20 | 2 |  |  |
|  | 21 | 1 |  |  |
|  | 28 | 1 |  |  |

*Note*: Physical Neglect Quality = Number of items endorsed for physical neglect from the Modified Multidimensional Neglectful Behavior Scale (MNBS-A; Straus et al., 1995); Physical Neglect Frequency = Frequency of physical neglect assessed through the MNBS-A; Emotional Neglect Quality = Number of items endorsed for emotional neglect from the MNBS-A; Emotional Neglect Frequency = Frequency of physical neglect assessed via the MNBS-A; Physical Abuse Quality = Number of items endorsed for physical abuse using a project-developed self-report measure of maltreatment; Physical Abuse Frequency = Frequency of physical abuse reported; Emotional Abuse Quality = Number of items endorsed for emotional abuse; Emotional Abuse Frequency = Frequency of emotional abuse reported; Sexual Abuse Quality = Number of items endorsed for sexual abuse; Sexual Abuse Frequency = Frequency of sexual abuse

Appendix B. Discrimination and Calibration Statistics across Specific Psychopathological Outcomes

| Variable | AUC | Intercept (CIL) | Slope | Emax | Spiegelhalter’s *z* | Spiegelhalter’s *p* |
| --- | --- | --- | --- | --- | --- | --- |
| Age 12 Anxiety (*n* = 17) | | | | | | |
| Cumulative Quality | **.68** (.55, .81)** | 0 (-2.98, 2.32) | 1 (.25, 1.66) | .03 (.01, .12) | .01 | .99 |
| Cumulative Frequency | **.65* (.53, .78)** | 0 (-4.40, 2.19) | 1 (-.11, 1.62) | .07 (.04, .24) | 0 | 1 |
| Emo. Neglect Quality | .55 (.40, .69) | 0 (-9.02, 3.92) | 1 (-1.28, 2.08) | .03 (.02, .18) | -.01 | 1 |
| Emo. Neglect Frequency | .58 (42, .71) | 0 (-11.4, 4.1) | 1 (-1.86, 2.12) | .03 (.03, .18) | 0 | 1 |
| Physical Abuse Quality | **.65* (.50, .79)** | 0 (-3.83, 2.18) | 1 (.03, 1.62) | .06 (.03, 1.62) | .02 | .98 |
| Physical Abuse Frequency | .62 (.47, .77) | 0 (-3.23, 1.82) | 1 (.17, 1.56) | .02^a^ | 0 | 1 |
| Emo. Abuse Quality | .63 (.49, .78) | 0 (-3.82, 2.31) | 1 (.05, 1.67) | .03 (.02, 13) | 0 | 1 |
| Emo. Abuse Frequency | **.64* (.50, .78)** | 0 (-4.46, 2.36) | 1 (-.12, 1.65) | .04 (.03, .19) | .01 | .99 |
| Sexual Abuse Quality | .59 (.45, .73) | 0 (-8.76, 4.17) | 1 (-1.18, 2.12) | .04 (.04, .13) | 0 | 1 |
| Age 12 Depression (*n* = 17) | | | | | | |
| Cumulative Quality | **.72** (.60, .84)** | 0 (-2.40, 1.99) | 1 (.42, 1.58) | .04 (.02, .15) | .02 | .99 |
| Cumulative Frequency | **.75** (.63, .87)** | 0 (-2.15, 1.49) | 1 (.47, 1.42) | .17 (.06, .44) | .07 | .94 |
| Emo. Neglect Quality | .59 (.44, .74) | 0 (-6.15, 3.18) | 1 (-.56, 1.85) | .02 (.01, .10) | 0 | 1 |
| Emo. Neglect Frequency | .61 (.47, .76) | 0 (-5.31, 2.85) | 1 (-.36, 1.76) | .08 (.04, .24) | .01 | .99 |
| Physical Abuse Quality | **.67* (.52, .81)** | 0 (-3.35, 2.06) | 1 (.18, 1.58) | .09 (.05, .15) | .03 | .98 |
| Physical Abuse Frequency | **.64* (.48, .80)** | 0 (-2.55, 1.58) | 1 (.36, 1.47) | .12 (.05, .34) | .01 | .99 |
| Emo. Abuse Quality | **.75** (.61, .89)** | 0 (-1.83, 1.37) | 1 (.55, 1.44) | .04 (.03, .17) | .04 | .97 |
| Emo. Abuse Frequency | **.77** (.63, .90)** | 0 (-1.74, 1.29) | 1 (.57, 1.36) | .06 (.04, .23) | .08 | .93 |
| Sexual Abuse Quality | .60 (.45, .74) | 0 (-9.85, 4.46) | 1 (-1.46, 2.21) | .05 (.03, .13) | 0 | 1 |
| Age 12 PTS (*n* = 18) | | | | | | |
| Cumulative Quality | **.75** (.63, .87)** | 0 (-1.95, 1.77) | 1 (.51, 1.57) | .05 (.02, .17) | .02 | .99 |
| Cumulative Frequency | **.75** (.64, .86)** | 0 (-1.97, 1.55) | 1 (.51, 1.43) | .19 (.07, .47) | .07 | .94 |
| Emo. Neglect Quality | .59 (.45, .73) | 0^a^ | 1^a^ | .02 | 0 | 1 |
| Emo. Neglect Frequency | .61 (.48, .75) | 0^a^ | 1^a^ | .05 | 0 | 1 |
| Physical Abuse Quality | **.70** (.57, .84)** | 0 (-2.46, 1.82) | 1 (.39, 1.52) | .10 (.06, .16) | .05 | .96 |
| Physical Abuse Frequency | **.68** (.53, .83)** | 0 (-2.39, 1.48) | 1 (.41, 1.43) | .05 (.05, .26) | .03 | .98 |
| Emo. Abuse Quality | **.70** (.57, .84)** | 0 (-2.52, 1.74) | 1 (.37, 1.53) | .02 (.02, .14) | .01 | .99 |
| Emo. Abuse Frequency | **.72** (.59, .86)** | 0 (-2.29, 1.50) | 1 (.44, 1.43) | .07 (.05, .19) | .03 | .98 |
| Sexual Abuse Quality | **.65* (.51, .80)** | 0 (-3.05, 1.95) | 1 (.24, 1.54) | .06 (.03, .23) | .03 | .98 |
| Age 12 Anger (*n* = 19) | | | | | | |
| Cumulative Quality | **.69** (.55, .82)** | 0 (-2.62, 2.24) | 1 (.35, 1.64) | .06 (.03, .12) | .01 | .99 |
| Cumulative Frequency | **.69** (.55, .82)** | 0 (-2.55, 1.84) | 1 (.37, 1.52) | .12 (.10, .24) | .05 | .96 |
| Emo. Neglect Quality | .61 (.47, .74) | 0 (-5.91, 2.88) | 1 (-.50, 1.83) | .04 (.02, .16) | 0 | 1 |
| Emo. Neglect Frequency | .62 (.49, .76) | 0 (-6.70, 2.44) | 1 (-.73, 1.70) | .06 (.05, .22) | 0 | 1 |
| Physical Abuse Quality | .55 (.41, .69) | 0 (-12.81, 5.16) | 1 (-2.35, 2.42) | .02 (.02, .10) | 0 | 1 |
| Physical Abuse Frequency | .53 (.39, .67) | 0 (-14.25, 4.04) | 1 (-2.79, 2.11) | .03 (.02, .16) | 0 | 1 |
| Emo. Abuse Quality | **.70** (.56, .84)** | 0 (-2.11, 1.45) | 1 (.46, 1.45) | .04 (.02, .16) | .02 | .99 |
| Emo. Abuse Frequency | **.69** (.55, .83)** | 0 (-2.23, 1.49) | 1 (.44, 1.44) | .05 (.03, .20) | .03 | .98 |
| Sexual Abuse Quality | .63 (.49, .76) | 0 (-7.18, 3.94) | 1 (-.86, 2.07) | .08 (.04, .17) | 0 | 1 |
| Age 12 Dissociation (*n* = 19) | | | | | | |
| Cumulative Quality | **.71** (.58, .84)** | 0 (-2.35, 2.04) | 1 (.39, 1.64) | .02 (.02, .16) | -.01 | 1 |
| Cumulative Frequency | **.67* (.55, .79)** | 0 (-3.42, 2.10) | 1 (.14, 1.61) | .07 (.04, .26) | .01 | .99 |
| Emo. Neglect Quality | .59 (.45, .73) | 0 (-6.58, 3.42) | 1 (-.70, 1.96) | .02 (.02, .10) | 0 | 1 |
| Emo. Neglect Frequency | .60 (.47, .74) | 0 (-7.40, 3.50) | 1 (-.95, 1.97) | .05 (.04, .20) | 0 | 1 |
| Physical Abuse Quality | **.66* (.52, .80)** | 0 (-3.43, 2.17) | 1 (.15, 1.60) | .09 (.04, .14) | .03 | .98 |
| Physical Abuse Frequency | .61 (.46, .75) | 0 (-4.33, 2.22) | 1 (-.11, 1.63) | .03 (.03, .18) | .01 | .99 |
| Emo. Abuse Quality | **.67* (.53, .80)** | 0 (-2.91, 2.09) | 1 (.26, 1.61) | .03 (.02, .11) | .01 | .99 |
| Emo. Abuse Frequency | **.69** (.56, .82)** | 0 (-2.76, 1.78) | 1 (.29, 1.52) | .05 (.04, .17) | .03 | .98 |
| Sexual Abuse Quality | **.63* (.50, .77)** | 0 (-4.52, 2.62) | 1 (-.15, 1.73) | .07 (.07, .21) | .01 | .99 |
| Age 16 Anxiety (*n* = 10) | | | | | | |
| Cumulative Quality | .62 (.44, .80) | 0 (-4.52, 2.62) | 1 (-.15, 1.73) | .07 (.07, .21) | .01 | .99 |
| Cumulative Frequency | .63 (.46, .79) | 0 (-14.62, 5.14) | 1 (-2.24, 2.25) | .03 (.02, .19) | 0 | 1 |
| Physical Neglect Quality | .50 (.32, .67) | 0 (-9.32, 172.8) | 1 (-1.07, 40.4) | .01 (.01, .05) | 0 | 1 |
| Physical Abuse Quality | .59 (.40, .78) | 0 (-16.37, 5.32) | 1 (-2.61, 2.27) | .04 (.03, .07) | 0 | 1 |
| Physical Abuse Frequency | .61 (.42, .80) | 0 (-25.7, 5.25) | 1 (-4.78, 2.27) | .04 (.04, .09) | 0 | 1 |
| Emo. Abuse Quality | **.69* (.54, .85)** | 0 (-5.86, 3.28) | 1 (-.26, 1.84) | .01 (.01, .11) | 0 | 1 |
| Emo. Abuse Frequency | **.73* (.58, .88)** | 0 (-6.15, 3.11) | 1 (-.32, 1.79) | .03 (.03, .14) | .01 | .99 |
| Sexual Abuse Quality | .57 (.38, .76) | 0 (-214.28, 6.05) | 1 (-46.16, 2.47) | .03 (.02, .15) | 0 | 1 |
| Sexual Abuse Frequency | .59 (.39, .78) | 0 (-404.54, 4.31) | 1 (-87.92, 2.10) | .04 (.03, .26) | 0 | 1 |
| Age 16 Depression (*n* = 19) | | | | | | |
| Cumulative Quality | **.65* (.52, .78)** | 0 (-3.16, 2.83) | 1 (.16, 1.85) | .04 (.01, .18) | -.01 | .99 |
| Cumulative Frequency | **.68** (.56, .80)** | 0 (-3.27, 2.03) | 1 (.15, 1.59) | .10 (.09, .24) | .03 | .98 |
| Physical Neglect Quality | .56 (.42, .70) | 0 (-7.96, 2.44) | 1 (-1.04, 1.69) | .03 (.03, .27) | 0 | 1 |
| Physical Abuse Quality | .62 (.47, .76) | 0 (-3.43, 2.00) | 1 (.13, 1.59) | .05 (.03, .19) | .01 | .99 |
| Physical Abuse Frequency | .61 (.46, .75) | 0 (-3.47, 2.22) | 1 (.10, 1.62) | .06 (.03, .19) | .02 | .99 |
| Emo. Abuse Quality | .61 (.48, .74) | 0 (-6.31, 3.59) | 1 (-.58, 2.01) | .02 (.02, .13) | 0 | 1 |
| Emo. Abuse Frequency | **.64* (.51, .77)** | 0 (-6.38, 3.33) | 1 (-.65, 1.91) | .04 (.04, .13) | .01 | 1 |
| Sexual Abuse Quality | .56 (.42, .71) | 0 (-7.90, 2.53) | 1 (-1.00, 1.76) | .07 (.06, .26) | -.02 | .99 |
| Sexual Abuse Frequency | .58 (.43, .72) | 0 (-7.79, 2.54) | 1 (-1.02, 1.72) | .02^a^ | .01 | 1 |
| Age 16 PTS (*n* = 8) | | | | | | |
| Cumulative Quality | .58 (.43, .72) | 0 (-31.37, 17.54) | 1 (-5.73, 4.90) | .02 (.02, .03) | 0 | 1 |
| Cumulative Frequency | .64 (.48, .80) | 0 (-41.17, 11.74) | 1 (-7.83, 3.60) | .02 (.02, .06) | 0 | 1 |
| Physical Neglect Quality | .51 (.31, .71) | 0 (-16.71, 322.56) | 1 (-2.54, 70.78) | .01 (.01, .05) | 0 | 1 |
| Physical Abuse Quality | .44 (.26, .63) | 0 (-5.33, 76.05) | 1 (-.08, 17.85) | 0 (0, .02) | 0 | 1 |
| Physical Abuse Frequency | .47 (.28, .65) | 0 (-4.18, 107.84) | 1 (.16, 24.78) | .01 (.01, .06) | 0 | 1 |
| Emo. Abuse Quality | .66 (.48, .84) | 0 (-10.64, 5.15) | 1 (-1.22, 2.18) | .02 (.01, .05) | 0 | 1 |
| Emo. Abuse Frequency | .70 (.52, .88) | 0 (-11.15, 3.89) | 1 (-1.28, 1.89) | .04 (.03, .10) | 0 | 1 |
| Sexual Abuse Quality | .55 (.34, .77) | 0 (-217.18, 6.28) | 1 (-44.46, 2.46) | .02 (.02, .15) | 0 | 1 |
| Sexual Abuse Frequency | .51 (.30, .72) | 0 (-454.04, 4.45) | 1 (-93.92, 2.11) | .04 (.03, .26) | -.01 | .99 |
| Age 16 Anger (*n* = 12) | | | | | | |
| Cumulative Quality | .48 (.31, .66) | 0 (-68.75, 46.14) | 1 (-15.05, 12.00) | .02 (.01, .10) | 0 | 1 |
| Cumulative Frequency | .49 (.31, .68) | 0 (-49.01, 6.69) | 1 (-10.34, 2.66) | .04 (.02, .13) | 0 | 1 |
| Physical Neglect Quality | .54 (.36, .72) | 0 (-91.25, 2.69) | 1 (-19.66, 1.74) | .03 (.03, .23) | -.02 | .99 |
| Physical Abuse Quality | .56 (.39, .74) | 0 (-16.64, 4.49) | 1 (-2.85, 2.14) | .02 (.01, .20) | 0 | 1 |
| Physical Abuse Frequency | .59 (.41, .77) | 0 (-18.35, 3.12) | 1 (-3.20, 1.79) | .02 (.02, .23) | 0 | 1 |
| Emo. Abuse Quality | .57 (.41, .73) | 0 (-33.23, 13.41) | 1 (-6.86, 4.24) | .02 (.02, .06) | 0 | 1 |
| Emo. Abuse Frequency | .58 (.40, .75) | 0 (-21.49, 6.85) | 1 (-3.96, 2.67) | .04 (.03, .12) | 0 | 1 |
| Sexual Abuse Quality | .46 (.31, .62) | 0 (-6.45, 153.92) | 1 (-.46, 38.00) | .01 (.01, .03) | 0 | 1 |
| Sexual Abuse Frequency | .48 (.32, .64) | 0 (-12.46, 950.23) | 1 (-1.90, 226.85) | .01 (.01, .06) | 0 | 1 |
| Age 16 Dissociation (*n* = 15) | | | | | | |
| Cumulative Quality | .63 (.47, .78) | 0 (-4.80, 3.78) | 1 (-.13, 2.04) | .04 (.01, .18) | -.01 | .99 |
| Cumulative Frequency | .64 (.50, .78) | 0 (-6.48, 3.17) | 1 (-.63, 1.85) | .05 (.04, .16) | .01 | .99 |
| Physical Neglect Quality | .59 (.42, .75) | 0 (-8.81, 2.43) | 1 (-1.18, 1.67) | .04 (.04, .23) | 0 | 1 |
| Physical Abuse Quality | .64 (.48, .79) | 0 (-4.22, 2.41) | 1 (-.03, 1.65) | .03 (.02, .16) | .01 | .99 |
| Physical Abuse Frequency | **.65* (.49, .81)** | 0 (-4.80, 2.25) | 1 (-.10, 1.61) | .03 (.03, .18) | .01 | .99 |
| Emo. Abuse Quality | .64 (.49, .79) | 0 (-5.51, 3.58) | 1 (-.37, 1.89) | .04 (.02, .08) | .01 | .99 |
| Emo. Abuse Frequency | **.66* (.52, .81)** | 0 (-6.09, 3.90) | 1 (-.50, 2.00) | .05 (.05, .10) | 0 | 1 |
| Sexual Abuse Quality | .56 (.40, .71) | 0 (-19.58, 6.27) | 1 (-3.80, 2.64) | .02 (.02, .14) | 0 | 1 |
| Sexual Abuse Frequency | .57 (.41, .73) | 0 (-22.05, 3.94) | 1 (-4.32, 2.03) | .05 (.04, .27) | 0 | 1 |
| Age 18 Anxiety (*n* = 27) | | | | | | |
| Cumulative Frequency | **.64* (.53, .74)** | 0 (-4.41, 2.62) | 1 (-.27, 1.82) | .04 (.03, .14) | .01 | .99 |
| Physical Abuse Frequency | .56 (.45, .68) | 0 (-6.57, 3.36) | 1 (-.94, 2.01) | .08 (.04, .15) | .01 | 1 |
| Emo. Abuse Quality | **.64* (.52, .75)** | 0 (-2.79, 2.04) | 1 (.23, 1.61) | .06 (.02, .12) | .02 | .98 |
| Emo. Abuse Frequency | **.64* (.52, .64)** | 0 (-2.94, 2.02) | 1 (.17, 1.63) | .07 (.03, .27) | .03 | .98 |
| Sexual Abuse Quality | .56 (.44, .68) | 0 (-6.00, 2.40) | 1 (-.75, 1.74) | .06 (.04, .23) | -.01 | .99 |
| Sexual Abuse Frequency | .55 (.43, .67) | 0 (-9.38, 2.73) | 1 (-1.71, 1.84) | .09 (.08, .32) | -.01 | .99 |
| Age 18 Depression (*n* = 45) | | | | | | |
| Cumulative Frequency | **.59* (.51, .68)** | 0 (-6.24, 4.41) | 1 (-1.14, 2.57) | .09 (.06, .13) | 0 | 1 |
| Physical Abuse Frequency | .52 (.43, .60) | 0 (-9.51, 4.66) | 1 (-2.31, 2.64) | .03 (.03, .16) | 0 | 1 |
| Emo. Abuse Quality | **.62** (.53, .71)** | 0 (-2.07, 1.75) | 1 (.29, 1.64) | .09 (.04, .16) | .02 | .98 |
| Emo. Abuse Frequency | **.61* (.52, .70)** | 0 (-2.42, 2.00) | 1 (.18, 1.69) | .09 (.05, .26) | .03 | .98 |
| Sexual Abuse Quality | .55 (.46, .64) | 0 (-4.93, 2.99) | 1 (-.68, 2.04) | .03 (.02, .15) | 0 | 1 |
| Sexual Abuse Frequency | .55 (.46, .64) | 0 (-11.41, 6.86) | 1 (-2.93, 3.40) | .09 (.06, .22) | 0 | 1 |
| Age 18 PTS (*n* = 86) | | | | | | |
| Cumulative Frequency | **.58* (.52, .65)** | 0 (-1.62, 1.51) | 1 (.26, 1.70) | .15 (.08, .28) | .03 | .98 |
| Physical Abuse Frequency | .56 (.49, .63) | 0 (-1.73, 1.51) | 1 (.23, 1.70) | .06 (.04, .25) | .01 | .99 |
| Emo. Abuse Quality | **.58* (.51, .65)** | 0 (-1.48, 1.23) | 1 (.31, 1.59) | .09 (.04, .20) | .02 | .98 |
| Emo. Abuse Frequency | **.58* (.51, .65)** | 0 (-1.51, 1.19) | 1 (.32, 1.55) | .07 (.05, .25) | .03 | .98 |
| Sexual Abuse Quality | .56 (.49, .63) | 0 (-1.35, 1.05) | 1 (.42, 1.50) | .05 (.04, .29) | -.02 | .98 |
| Sexual Abuse Frequency | .55 (.48, .62) | 0 (-1.62, 1.33) | 1 (.26, 1.61) | .07 (.07, .37) | .02 | .98 |
| Age 18 Anger (*n* = 49) | | | | | | |
| Cumulative Frequency | **.62** (.54, .70)** | 0 (-3.29, 2.52) | 1 (-.15, 1.93) | .12 (.07, .16) | .01 | .99 |
| Physical Abuse Frequency | **.61** (.52, .70)** | 0 (-2.27, 1.71) | 1 (.20, 1.62) | .12 (.06, .24) | .03 | .98 |
| Emo. Abuse Quality | **.64** (.56, .73)** | 0 (-1.43, 1.25) | 1 (.51, 1.46) | .15 (.10, .20) | .06 | .95 |
| Emo. Abuse Frequency | **.63** (.54, .72)** | 0 (-1.92, 1.84) | 1 (.33, 1.67) | .17 (.14, .23) | .04 | .97 |
| Sexual Abuse Quality | .58 (.49, .67) | 0 (-3.00, 2.23) | 1 (-.06, 1.78) | .08 (.05, .19) | .01 | .99 |
| Sexual Abuse Frequency | .58 (.49, .67) | 0 (-3.41, 2.71) | 1 (-.20, 1.99) | .14 (.12, .30) | .02 | .99 |
| Age 18 Dissociation (*n* = 51) | | | | | | |
| Cumulative Frequency | **.66** (.58, .74)** | 0 (-1.67, 1.49) | 1 (.40, 1.55) | .15 (.06, .26) | .06 | .96 |
| Physical Abuse Frequency | .57 (.49, .66) | 0 (-3.21, 2.44) | 1 (-.16, 1.92) | .08 (.04, .20) | .01 | .99 |
| Emo. Abuse Quality | **.61** (.53, .70)** | 0 (-1.76, 1.53) | 1 (.36, 1.57) | .09 (.04, .17) | .03 | .98 |
| Emo. Abuse Frequency | **.63** (.55, .72)** | 0 (-1.90, 1.60) | 1 (.32, 1.59) | .13 (.07, .21) | .04 | .97 |
| Sexual Abuse Quality | **.61** (.52, .70)** | 0 (-1.42, 1.09) | 1 (.48, 1.42) | .08 (.05, .27) | -.01 | .99 |
| Sexual Abuse Frequency | **.61** (.52, .70)** | 0 (-1.42, 1.36) | 1 (.49, 1.50) | .09 (.09, .38) | .05 | .96 |

*Note*: Bold = AUCs above 0.64 threshold; CIL = Calibration-in-the-Large; Cumulative Quality = Total number of maltreatment subtypes endorsed at Baseline (Age 12); Cumulative Frequency = Sum of the frequency of each subtype divided by the number of items used to assess each subtype, respectively, endorsed at Baseline; Physical Neglect Quality = Number of items endorsed for physical neglect from the Modified Multidimensional Neglectful Behavior Scale (MNBS-A; Straus et al., 1995); Physical Neglect Frequency = Frequency of physical neglect assessed through the MNBS-A; Emo. Neglect Quality = Number of items endorsed for emotional neglect from the MNBS-A; Emo. Neglect Frequency = Frequency of physical neglect assessed via the MNBS-A; Physical Abuse Quality = Number of items endorsed for physical abuse using a project-developed self-report measure of maltreatment; Physical Abuse Frequency = Frequency of physical abuse reported; Emo. Abuse Quality = Number of items endorsed for emotional abuse; Emo. Abuse Frequency = Frequency of emotional abuse reported; Sexual Abuse Quality = Number of items endorsed for sexual abuse; Sexual Abuse Frequency = Frequency of sexual abuse; Age 12 Psychopathology = Diagnostic status reflecting clinically significant levels of depression, anxiety, anger, post-traumatic stress, or dissociation at Baseline on the Trauma Symptom Checklist for Children (TSCC; Briere, 1996); Age 16 Psychopathology = Diagnostic status reflecting clinically significant levels of depression, anxiety, anger, post-traumatic stress, or dissociation at age 16 on the TSCC (Briere, 1996); Age 18 Psychopathology = Diagnostic status reflecting clinically significant levels of depression, anxious arousal, anger/irritability, intrusive experiences, defensive avoidant, or dissociation at age 16 on the Trauma Symptom Inventory (TSI; Briere, 1996).

**p* < .05; ***p* < .01

^a^ Results indicating impermissible solutions for confidence intervals

Appendix C. Logistic Regression Model Outcomes

|  | B | SE | Wald | Exp(B) | Exp(B) 95% CI |
| --- | --- | --- | --- | --- | --- |
| Age 12 Psychopathology | | | | | |
| Cumulative Quality | .52 | .12 | 19.65** | 1.69 | 1.34 – 2.13 |
| Cumulative Frequency | .62 | .15 | 18.13** | 1.87 | 1.40 – 2.49 |
| Phys. Neg. Quality | .18 | .20 | .80 | 1.20 | .81 – 1.79 |
| Phys. Neg. Frequency | .19 | .16 | 1.46 | 1.21 | .89 – 1.66 |
| Emo. Neg. Quality | .23 | .10 | 4.96* | 1.25 | 1.03 – 1.53 |
| Emo. Neg. Frequency | .12 | .06 | 4.38* | 1.28 | 1.01 – 1.26 |
| Phys. Abuse Quality | .35 | .13 | 7.74** | 1.42 | 1.11 – 1.81 |
| Phys. Abuse Frequency | .21 | .05 | 14.36** | 1.23 | 1.11 – 1.37 |
| Emo. Abuse Quality | .24 | .05 | 22.72** | 1.27 | 1.15 – 1.40 |
| Emo. Abuse Frequency | .11 | .02 | 25.68** | 1.12 | 1.07 – 1.17 |
| Sex. Abuse Quality | .20 | .10 | 4.10* | 1.22 | 1.01 – 1.49 |
| Sex. Abuse Frequency | .07 | .04 | 3.29 | 1.07 | .99 – 1.16 |
| Age 16 Psychopathology | | | | | |
| Cumulative Quality | .25 | .13 | 3.99* | 1.29 | 1.01 – 1.65 |
| Cumulative Frequency | .47 | .17 | 8.01** | 1.59 | 1.15 – 2.20 |
| Phys. Neg. Quality | .43 | .17 | 6.55* | 1.54 | 1.11 – 2.14 |
| Phys. Neg. Frequency | .14 | .18 | .59 | 1.14 | .81 – 1.62 |
| Emo. Neg. Quality | .16 | .11 | 1.90 | 1.17 | .94 – 1.46 |
| Emo. Neg. Frequency | .07 | .07 | 1.28 | 1.08 | .95 – 1.22 |
| Phys. Abuse Quality | .29 | .13 | 4.89* | 1.34 | 1.03 – 1.74 |
| Phys. Abuse Frequency | .15 | .06 | 5.43* | 1.16 | 1.02 – 1.31 |
| Emo. Abuse Quality | .14 | .06 | 5.99* | 1.15 | 1.03 – 1.29 |
| Emo. Abuse Frequency | .06 | .03 | 4.42* | 1.06 | 1.00 – 1.12 |
| Sex. Abuse Quality | .26 | .10 | 6.92** | 1.30 | 1.07 – 1.58 |
| Sex. Abuse Frequency | .10 | .04 | 7.11** | 1.10 | 1.03 – 1.19 |
| Age 18 Psychopathology | | | | | |
| Cumulative Quality | .09 | .08 | 1.44 | 1.10 | .94 – 1.28 |
| Cumulative Frequency | .24 | .12 | 3.91* | 1.28 | 1.00 – 1.62 |
| Phys. Neg. Quality | -.05 | .17 | .09 | .95 | .69 – 1.31 |
| Phys. Neg. Frequency | .003 | .13 | .001 | 1.003 | .77 – 1.30 |
| Emo. Neg. Quality | -.05 | .09 | .28 | .96 | .81 – 1.13 |
| Emo. Neg. Frequency | -.04 | .06 | .45 | .96 | .87 – 1.07 |
| Phys. Abuse Quality | .16 | .10 | 2.83 | 1.18 | .97 – 1.42 |
| Phys. Abuse Frequency | .10 | .05 | 5.09* | 1.11 | 1.01 – 1.21 |
| Emo. Abuse Quality | .09 | .04 | 5.74* | 1.10 | 1.02 – 1.81 |
| Emo. Abuse Frequency | .04 | .02 | 4.82* | 1.04 | 1.01 – 1.08 |
| Sex. Abuse Quality | .21 | .07 | 8.58** | 1.23 | 1.07 – 1.41 |
| Sex. Abuse Frequency | .07 | .03 | 6.09* | 1.07 | 1.01 – 1.13 |

*Note*: Models reflect independent logistic regression models. All models included gender, race/ethnicity, and recruitment site as covariates, while Age 16 and Age 18 psychopathology models additionally controlled for Age 12 psychopathology. Wald = Wald chi-square test; Exp(B) = Exponentiated coefficient (i.e., odds ration); Cumulative Quality = Total number of maltreatment subtypes endorsed at Baseline (Age 12); Cumulative Frequency = Sum of the frequency of each subtype divided by the number of items used to assess each subtype, respectively, endorsed at Baseline; Phys. Neg. Quality = Number of items endorsed for physical neglect from the Modified Multidimensional Neglectful Behavior Scale (MNBS-A; Straus et al., 1995); Phys. Neg. Frequency = Frequency of physical neglect assessed through the MNBS-A; Emo. Neg. Quality = Number of items endorsed for emotional neglect from the MNBS-A; Emo. Neg. Frequency = Frequency of physical neglect assessed via the MNBS-A; Phys. Abuse Quality = Number of items endorsed for physical abuse using a project-developed self-report measure of maltreatment; Phys. Abuse Frequency = Frequency of physical abuse reported; Emo. Abuse Quality = Number of items endorsed for emotional abuse; Emo. Abuse Frequency = Frequency of emotional abuse reported; Sex. Abuse Quality = Number of items endorsed for sexual abuse; Sex. Abuse Frequency = Frequency of sexual abuse; Age 12 Psychopathology = Diagnostic status reflecting clinically significant levels of depression, anxiety, anger, post-traumatic stress, or dissociation at Baseline on the Trauma Symptom Checklist for Children (TSCC; Briere, 1996). Age 16 Psychopathology = Diagnostic status reflecting clinically significant levels of depression, anxiety, anger, post-traumatic stress, or dissociation at age 16 on the TSCC (Briere, 1996); Age 18 Psychopathology = Diagnostic status reflecting clinically significant levels of depression, anxious arousal, anger/irritability, intrusive experiences, defensive avoidant, or dissociation at age 18 on the Trauma Symptom Inventory (TSI; Briere, 1996).

**p* < .05; ***p* < .01
